# The Impact of a Dedicated Research Education Month for Anesthesiology Residents

**DOI:** 10.1155/2015/623959

**Published:** 2015-01-13

**Authors:** Robert E. Freundlich, Jessica W. Newman, Kevin K. Tremper, Jill M. Mhyre, Sachin Kheterpal, Theodore J. Sanford, Alan R. Tait

**Affiliations:** ^1^Department of Anesthesiology, University of Michigan Medical School, Ann Arbor, MI 48105, USA; ^2^Department of Anesthesiology, University of Arkansas, Little Rock, AR 72205, USA

## Abstract

An educational intervention was implemented at the University of Michigan starting in 2008, in which anesthesiology interns complete a dedicated month-long didactic rotation in evidence-based medicine (EBM) and research methodology. We sought to assess its utility. Scores on a validated EBM test before and after the rotation were compared and assessed for significance of improvement. A survey was also given to gauge satisfaction with the quality of the rotation and self-reported improvement in understanding of EBM topics. Fourteen consecutive interns completed the research rotation during the study period. One hundred percent completed both the pre- and postrotation test. The mean pretest score was 7.78 ± 2.46 (median = 7.5, 0–15 scale, and interquartile range 7.0–10.0) and the mean posttest score was 10.00 ± 2.35 (median = 9.5, interquartile range 8.0–12.3), which represented a statistically significant increase (*P* = 0.011, Wilcoxon signed-rank test). All fourteen of the residents “agreed” or “strongly agreed” that they would recommend the course to future interns and that the course increased their ability to critically review the literature. Our findings demonstrate that this can be an effective means of improving understanding of EBM topics and anesthesiology research.

## 1. Introduction

While most medical schools teach EBM in one form or another, it has been our experience that the quality of this preparation varies between residents. Furthermore, the relevance to anesthesiology practice may be limited, since most examples from medical school courses are drawn from the general medical disciplines. A 2004 meta-analysis comparing various studies reporting EBM learning in medical education suggested that postgraduate medical education in EBM topics may be particularly useful in teaching critical appraisal skills. The authors attributed this value to a general desire among medical postgraduates to learn for the sake of improved patient care, as opposed to the undergraduate motivation of higher test scores [1].

There have been several reports of successful teaching methods, such as regular journal clubs [2], in anesthesiology residency programs to increase critical thinking and appraisal of anesthesiology research. Other fields have also reported the successful adoption of dedicated research rotations in their residencies, notably internal medicine [3] and pediatrics [4]. In addition, residency programs have reported regularly setting aside dedicated time for EBM teaching [5–7].

To the best of our knowledge, we have implemented the first dedicated month-long EBM course in an anesthesiology residency program. We sought to assess whether participation in the research rotation would lead to quantitative and self-reported improved understanding of EBM topics among our intern class and whether residents were satisfied with their experience.

## 2. Materials and Methods

*Informed Consent*. This study was granted a waiver of informed consent under the educational exemption by the University of Michigan Institutional Review Board (Ann Arbor, Michigan).

### 2.1. Prospective Cohort and Questionnaire

In 2008, due to a perceived weakness in resident understanding and appreciation of evidence-based medicine (EBM) topics and as a vehicle to offer early research exposure to residents, a month-long dedicated research rotation was implemented at the University of Michigan. All categorical anesthesiology Postgraduate Year-1 (PGY-1) residents now take the course as a required part of their intern curriculum. During this month, participants complete reading assignments in preparation for small group discussions on various EBM topics (Table 1). Residents also review an assigned journal article and present it during a departmental journal club. Lastly, under the guidance of a research mentor, residents design and present an original research proposal. In preparing the proposal, they must develop an answerable research question, devise a hypothesis, and perform a focused literature review. The resulting proposal is presented to the Department Chair for review. To evaluate the impact of the research rotation, a prospective cohort study was conducted on a consecutive group of PGY-1 interns during the 2011-12 academic year. This present study was the result of a research rotation proposal from two of the rotation's participants (Robert E. Freundlich and Jessica W. Newman). All interns who participated in the research rotation were eligible for inclusion. The English version of the Berlin EBM Questionnaire was used to assess the value of the rotation [3, 8, 9]. The Berlin Questionnaire consists of two distinct sets (A and B) of 15 multiple-choice questions, in which participants apply statistical concepts to resolve theoretical patient care issues. Each participant completed one of the sets (set A or set B) before and the other after the intervention. All questions were weighted equally when determining the score and for statistical analysis.

The Berlin Questionnaire focuses on the following domains of EBM: ability to understand and critically appraise evidence, relating a clinical problem to a clinical question, identifying relevant study design, and using basic statistics to solve specific patient issues [10]. Furthermore, the Berlin Questionnaire has proven content validity and internal consistency. The Berlin Questionnaire has been used in prior studies involving medical students [11], internal medicine residents [3], and other health care professionals [8] to evaluate the impact of educational interventions on EBM knowledge and skills.

A research assistant distributed the questionnaire on the first and last day of the course. Faculty lecturers did not have access to the questionnaire and the results were not used in the residents' course evaluation. The primary outcome measure was the mean change in scores on the Berlin Questionnaire before and after the research month.

### 2.2. Survey

At the conclusion of the research rotation residents were required to fill out a survey to evaluate the course (Table 2). The survey included ten questions with a five-point Likert response scale and a narrative comments' section. Questions covered the organization of the course, lecture topics, and perceived increase in knowledge.

### 2.3. Statistical Analysis

A pilot study was performed in 2011 to calculate statistical power and for sample size determination. Five residents enrolled in the research rotation took the Berlin Questionnaire before and after the course.

Residents' scores on the Berlin Questionnaire before and after the course were recorded. The Wilcoxon signed-rank test was used to analyze paired data. The mean and standard deviations for the answers to the survey were analyzed and the statistical difference of the mean was calculated from a neutral response using the one-sample *t*-test. Data are presented as mean, standard deviation, median, and interquartile range (IQR). Statistical significance was accepted at *P* < 0.05.

The mean initial score during the pilot study was 8.0/15 (*σ* = 1.2) and the mean final score during the pilot study was 10.8/15 (*σ* = 2.3). Based on these findings, we estimated that we would need 9 residents to participate in order to generate a power of 0.9 to detect a difference in scores of at least this large with an alpha of 0.05 (2-sided).

## 3. Results

Fourteen consecutive participants completed the pre- and postcourse test during the 2011-12 academic year, including the five included in the pilot study. All participants completed both the pre- and postcourse test. The mean baseline score was 7.78/15 (*σ* = 2.46, median 7.5, and IQR 7.0–10.0) and the mean postcourse score was 10.0/15 (*σ* = 2.35, median 9.5, and IQR 8.0–12.3), which represented a statistically significant increase (*P* = 0.011).

Results from the survey are described in Table 2. The response rate for the survey was 100%. All of the residents stated that they either “agree” or “strongly agree” that they would recommend the course to future interns and that the course increased their ability to critically review the literature.

Residents have actively pursued their intern research projects and seen them through to completion. To date, several of these projects have been published [12, 13] and others are currently under journal review.

## 4. Discussion

We sought to prospectively evaluate the impact of a dedicated, month-long research rotation in an anesthesiology residency program. This educational intervention successfully improved resident performance on a standardized metric and resulted in high self-reported improvement in comprehension of EBM topics. We anticipate that the skills and knowledge obtained in this course will advance our residents' abilities to critically review the literature and use EBM in their own practice and in their clinical decision-making.

The research rotation was implemented using existing resources. Certain aspects of its structure may have influenced its success. An intern class at the University of Michigan has 24 residents. To ensure high faculty-to-participant ratios, the course is offered eight times per year to only three residents at a time. Didactic topics (Table 1) are generally presented in a discussion-based format, although some topics are also reviewed in supplemental online lectures. We believe that this could serve as a model for teaching EBM at other anesthesiology residency programs. Furthermore, the program could be applied to other specialties, medical students, or other health professionals, such as nurse anesthetists and anesthesiology assistants.

Although this was not our primary aim in implementing the research rotation, it is interesting to note that 11 participants felt that the course increased their interest in pursuing research. As has been discussed elsewhere, there is considerable appeal in finding successful means of increasing resident training and interest in anesthesiology research [14–16].

There are several limitations to our intervention that deserve further discussion. Although we found an increased score on a validated EBM questionnaire, we are unable, at this time, to determine if this increase will ultimately translate into improved patient outcomes, better utilization of the literature, and long-term understanding of EBM topics. Given that this test was administered immediately after the end of the course's completion, we were unable to assess long-term retention of knowledge gained. In addition, given the relatively small sample size, we were also unable to conduct analyses to determine if improvement on the test was global or limited to certain EBM topics. It is possible that some programs may find it difficult to place residents in a nonclinical rotation, given patient care needs at their institution. Finally, the single center nature of this intervention may limit the generalizability of our findings. Future studies will seek to address many of these issues.

## 5. Conclusions

In conclusion, results of this study suggest that implementation of a month-long, dedicated intern research rotation successfully increased understanding of EBM topics in a cohort of anesthesiology residents. Future work will seek to better refine how we can improve resident understanding of topical areas as we continue to offer the course to our residents and track their performance using the Berlin Questionnaire. We believe that this course has had a significant impact on our resident education and could be successfully applied elsewhere.

## Figures and Tables

**Table 1:** Didactic topics in the intern research rotation.

1	Research committee and ongoing research
2	Descriptive statistics
3	Selecting a statistical test
4	Hypothesis testing
5	Measures of association and effect size and logistic regression
6	Cohort and observational studies
7	Designing and evaluating randomized controlled trials
8	Assessing randomized controlled trials
9	Diagnosis and screening
10	Meta-analysis and systemic review
11	The manuscript process
12	Evidence-based practice
13	Reading and presenting a research study
14	Introduction to the institutional review board (IRB)

**Table 2:** Resident final course evaluation survey.

	Strongly disagree	Disagree	Neither agree nor disagree	Agree	Strongly agree
(1) The research rotation was well organized	0/14	0/14	0/14	7/14	7/14
(2) The goals/objectives of the rotation were met	0/14	0/14	0/14	4/14	10/14
(3) The instructors demonstrated a thorough grasp of the material	0/14	0/14	0/14	3/14	11/14
(4) The lecture topics were comprehensive	0/14	0/14	0/14	5/14	9/14
(5) The lecture topics were relevant	0/14	0/14	0/14	3/14	11/14
(6) The course increased my understanding of research	0/14	0/14	0/14	2/14	12/14
(7) The course increased my interest in pursuing research	0/14	0/14	3/14	1/14	10/14
(8) The course increased my ability to critically review the literature	0/14	0/14	0/14	4/14	10/14
(9) I would recommend this course to future interns	0/14	0/14	0/14	3/14	11/14
(10) I would have preferred a clinical rotation	6/14	6/14	0/14	0/14	2/14
(11) Suggestions for improvement					
(12) Additional comments					
